# Supplementary material for: ABCB1 Does Not Require the Side-Chain Hydrogen-Bond Donors Gln347, Gln725, Gln990 to Confer Cellular Resistance to the Anticancer Drug Taxol
Source: Int J Mol Sci. 2021 Aug 9;22(16):8561. doi: 10.3390/ijms22168561 (PMC8395328; doi:10.3390/ijms22168561)
Supplement: Supplementary file 1 [file ijms-22-08561-s001.zip › ijms-1259042-supplementary.pdf]

Supplementary Materials

(a)

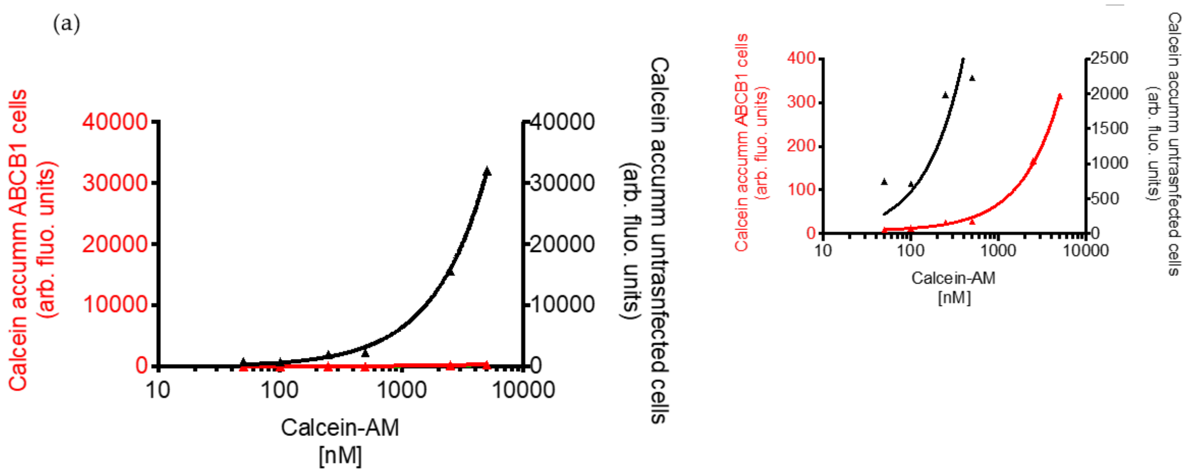

(b)

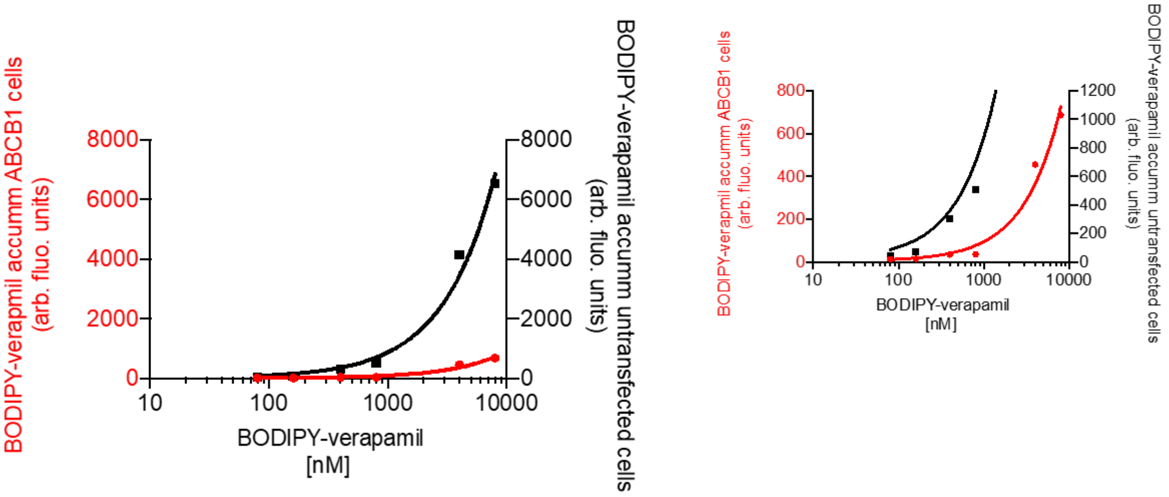

(c)

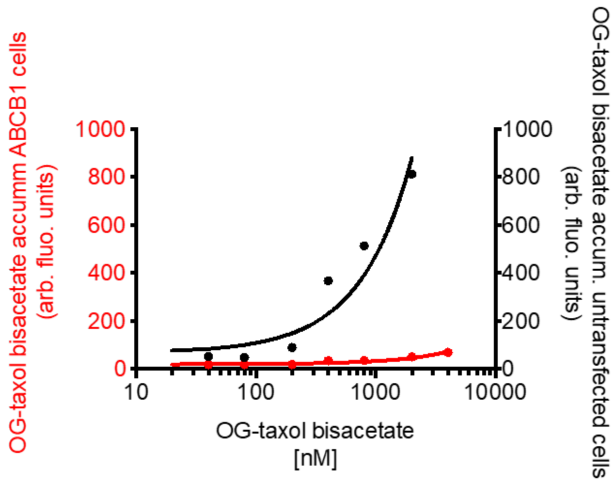

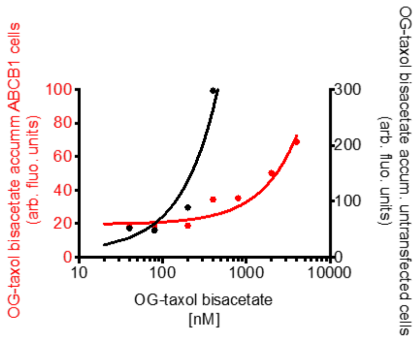

Figure S1. Titration of transport substrates for development of flow cytometry assay. HEK293T cells were transiently transfected with pABCB1. Cells were labelled with the anti-ABCB1 primary 4E3 antibody as described in the methods. The red-fluorescent secondary antibody was added and the cells divided into 100 µl aliquots to which were added increasing concentrations of green-fluorescent transport substrate. After 20 minutes at 37 °C the cells were recovered by gentle centrifugation, washed and resuspended in transport buffer for flow cytometry. Ten thousand cells of normal size and granularity were analysed for antibody binding to distinguish the ABCB1-expressing from the untransfected cells. The level of transport substrate uptake by the two populations was plotted against the final concentration of transport substrate added to the cell samples using Graphpad Prism version 8. The transport substrate content of the ABCB1-expressing (transfected) cells is shown in red (left Y axis) and the non-expressing (untransfected) cells in black (right Y axis) for: (**a**) Calcein-AM; (**b**) BODIPY-verapamil; (**c**) OREGON GREEN Taxol bisacetate. The graph on the left shows the full range of concentrations for each drug with the same Y-axis scale for drug content of the two cell populations. For the graph on the right, the Y-axes have been scaled appropriate to the two cell populations to focus on the drug concentration at which accumulation by the ABCB1-expressing cells (the red curve) begins to increase. The concentration of drug used for subsequent experiments was determined as the point at which this red curve begins to accumulate transport substrate indicating that transporter density at the plasma membrane is becoming saturated. This was deemed to be 500 nM for Calcein-AM, 800 nM for BODIPY-verapamil and 400 nM for OREGON GREEN-Taxol bisacetate. These concentrations also provide good discrimination between the ABCB1-expressing and non-expressing cells in the population but are minimal thus limiting any possible cytotoxic effects of the verapamil or taxol derivatives during the assay.

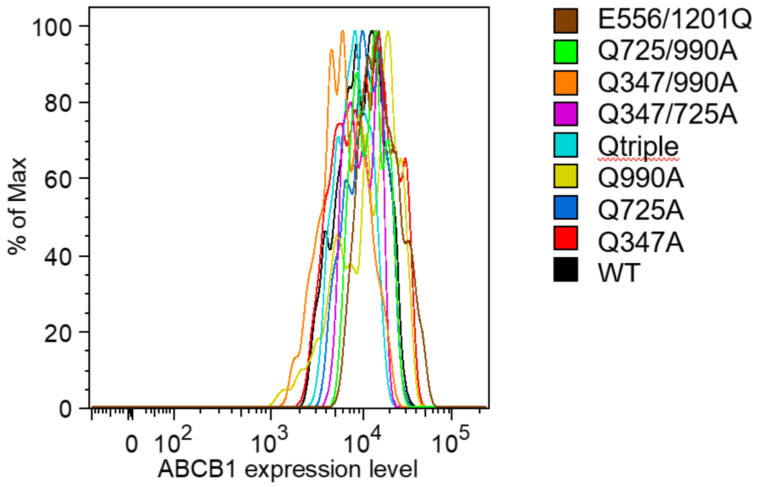

Figure S2. Confirmation of equivalent levels of ABCB1 at the plasma membrane ensured that any differences in transport activity were due to ABCB1 functionality rather than the transporter expression level. Histogram of the 4E3-positive populations showing significant overlap in red fluorescence of wild-type ABCB1 and each of the mutant transporters.
